# Effects of Interactions between Feeding Patterns and the Gut Microbiota on Pig Reproductive Performance

**DOI:** 10.3390/ani14182714

**Published:** 2024-09-19

**Authors:** Mingyu Wang, Jiaqi Yue, Guangquan Lv, Yaxin Wang, Ao Guo, Zhe Liu, Taiyong Yu, Gongshe Yang

**Affiliations:** Key Laboratory of Animal Genetics, Breeding and Reproduction of Shaanxi Province, Laboratory of Animal Fat Deposition & Muscle Development, College of Animal Science and Technology, Northwest A&F University, Yangling 712100, China; wangmingyu@nwafu.edu.cn (M.W.); yuejiaqi@nwafu.edu.cn (J.Y.); lvguangquan@nwsuaf.edu.cn (G.L.); wangyaxin1999@nwafu.edu.cn (Y.W.); guoaoyavis@nwafu.edu.cn (A.G.); liuzhesci@126.com (Z.L.)

**Keywords:** feeding mode, performance of reproduction, gut microbiota, sows in late gestation

## Abstract

**Simple Summary:**

With the development of technology and science, automated farming methods have gradually begun to replace traditional manual feeding methods, offering advantages such as precise feeding and reduced labor costs. However, there is still a lack of extensive research on the effects of different feeding methods on the reproductive performance of pigs and the diversity of their intestinal microbiota. This study analyzed the reproductive performance of Yorkshire pigs fed the same diet under different feeding methods, and further explored the diversity of their intestinal microbiota, aiming to elucidate the interactions between feeding methods, intestinal microbiota, and reproductive phenotypes, in order to understand the impact of the interaction between feeding methods and intestinal microbiota on host reproductive performance. This study aims to provide a certain theoretical and experimental basis for the promotion of automated feeding modes.

**Abstract:**

The feeding mode is an important factor affecting the reproductive performance of pigs. The composition and expression of the intestinal microbiota are closely related to the physiological and biochemical indicators of animals. Therefore, to explore the impact of different feeding patterns on the reproductive performance of pigs, this study collected reproductive performance data from 1607 Yorkshire pigs raised under different feeding patterns and conducted a fixed-effect variance analysis. Among them, 731 were in the artificial feeding (AM) group and 876 were in the feeding station feeding (SM) group. Additionally, 40 Yorkshire sows in the late gestation period were randomly selected from each feeding mode for intestinal microbiota analysis. The results of the analysis showed that, in the AM group, both the number of birth deformities (NBD) and the number of stillbirths (NSB) were significantly greater than they were in the SM group (*p* < 0.05). Additionally, the total number born (TNB) in the AM group was significantly lower than that in the SM group (*p* < 0.05). The results of the intestinal microbiota analysis revealed that at the phylum level, there were significant differences in nine bacterial taxa between the AM and SM groups (*p* < 0.05). At the genus level, the abundance of a variety of beneficial bacteria related to reproductive performance in the SM group was significantly greater than that in the AM group. Finally, fecal metabolomic analysis revealed that the contents of butyric acid, isovaleric acid, valeric acid, and isobutyric acid, which are associated with reproductive performance, in the feces of sows in the SM group were significantly higher than those in the AM group (*p* < 0.05). These results indicate that different feeding methods can affect the gut microbiota composition of Yorkshire pigs and further influence the reproductive performance of pigs through the gut microbiota–metabolic product pathway. The results of this study provide valuable insights for further exploring the relationships between feeding modes, intestinal microbial composition, and host phenotypes.

## 1. Introduction

Until the 1960s, pig production was typically based on numerous traditional smallholder family rearing systems, with approximately 4000 m^2^ of pasture access per 20 pigs. During the last half of the 20th century, changes in animal farming modified the applied housing practices and management, and production was converted from extensive to intensive commercialized facilities [1]. The transition from extensive to intensive housing has induced great changes in pig husbandry. For example, piglets are naturally weaned at approximately 17 weeks of age [2], while under intensive housing conditions, they are commonly weaned prematurely at approximately 4–5 weeks of age. Furthermore, the feeding method has gradually changed from manual feeding to automated feeding station feeding. Among these methods, artificial feeding is characterized by a high degree of flexibility, allowing breeders to adjust and manage it according to specific circumstances and demands [3]. Additionally, it enables the effective monitoring of the pigs’ health status. Feeding pigs through feeding stations has been found to effectively reduce production costs and enhance the utilization efficiency of feed, water equipment, and other resources [4]. However, no studies have examined the impact of different feeding methods on the reproductive performance of pigs.

The reproductive performance of animals is intricately linked to economic benefits, making the improvement of animal reproductive performance a significant research focus within the breeding industry. Emerging studies have revealed the involvement of gut microbes in the regulation of animal reproductive performance. A study conducted by Xin et al. demonstrated that the administration of *Lactobacillus johnsonii BS15* resulted in a significant improvement in the daily weight gain and diarrhea index of piglets [5]. Additionally, this intervention had positive effects on the growth, development ability, and disease resistance of piglets to some extent [5]. In addition, a study conducted by Sun et al. revealed that sows experience metabolic syndrome and undergo significant alterations in the composition of their intestinal microbiota during late gestation and lactation. These changes have implications for sow performance and the health of piglets [6].

It is widely recognized that various factors, including the environment, diet, and genetic background, can influence the composition of the intestinal microbiota in animals, subsequently impacting their phenotypic traits [7]. While different feeding methods are known to affect the reproductive performance of pigs, the specific mechanisms through which these impacts occur remain unclear. Consequently, this study aimed to investigate potential differences in reproductive performance by employing various feeding methods on Yorkshire pigs. The ultimate objective of this study was to provide valuable insights into scientific and precision breeding techniques for pigs by determining the potential microbial communities linked to differences in feeding methods and their impact on pig reproductive performance.

## 2. Materials and Methods

All the animal studies were approved by the Institutional Animal Care and Use Committee of Northwest A&F University (approval number: NWAFU-314021167). All operations were carried out in accordance with the university guidelines for animal research. All pigs were cared for and sacrificed following the guidelines of the Institutional Animal Care and Use Committee of Northwest A&F University (Yangling, China).

### 2.1. Animals and Reproductive Performance Measurements

This study conducted an initial statistical analysis of breeding records for 6431 litters from 1607 Yorkshire pigs at a prominent core breeding facility in Shaanxi Province, China. The data analysis covered the period from 2016 to 2023, during which different feeding modes were utilized. Specifically, 876 litters were raised under the feeding station mode, while 731 litters were raised under the artificial feeding mode. It should be noted that all sows measured in this study were at parities 4 to 6. The sows in the SM group were fed using fully automatic electronic feeders produced by Osborne Industries in the United States. The sows in the AM group were manually fed at 8 a.m. and 4 p.m. daily, with an average daily feed intake of 3.4 kg in the summer and 3.6 kg in the winter. The statistical indicators of reproductive performance included the total number born (TNB), number born alive (NBA), litter birth weight (LBW), gestation length (GL), number of weaned piglets (NW), number of piglets born weak (NBW), number of stillbirths (NSB), number of mummies (MUM), and number of birth deformities (NBD). Subsequently, referring to the pedigree records on the farm, we selected 80 sows in good health, of similar size, in their fifth parity, and from different feeding systems with distant genetic relationships for fecal sample collection to evaluate the gut microbial diversity of Yorkshire sows. (feeding station feeding mode: n = 40; artificial feeding mode: n = 40). Finally, seven individuals were randomly selected from each different feeding mode for targeted metabolomic profiling. All sows used in the experiment were housed in different pens within the same pig house, which was equipped with temperature and humidity control facilities such as water curtains. The temperature in the pig house was maintained at 20–23 °C, and the humidity was kept between 65% and 80%. All experimental animals were fed the same diet composed of corn and soybeans. (Table 1). All the measured data were corrected and analyzed by means of SPSS 22.0. The estimation of genetic parameters for nine reproductive traits was conducted using the repeatability model implemented in ASREML (version 4.10) software. Additionally, the analysis of fixed-effect variances was performed using the GLM in SAS (version 9.2) software.

### 2.2. Gut Mikrobiota Detection

At the conclusion of the experimental period, fecal samples were obtained from all the pigs through rectal massage and subsequently preserved in liquid nitrogen. A QIAamp DNA Stool Mini Kit (Qiagen, Hamburg, Germany) was used to extract total genomic DNA following the manufacturer’s protocols [8]. The concentration and quantity of DNA were measured using a NanoDrop 1000 spectrophotometer (NanoDrop, Wilmington, DE, USA), and the DNA was subsequently diluted to a final concentration of 1 ng/μL using sterile water. 16S ribosomal RNA (rRNA) gene sequencing was performed by Shanghai Sangon Biotech on the Illumina HiSeq 2500 platform (The equipment was purchased from Illumina, Inc. in San Francisco, CA, USA). The specific V3-V4 regions of the 16S rRNA genes were amplified using the designated primers (forward: GTGCCAGCMGCCGCGGTAA; reverse: GGACTACHVGGGTWTCTAAT) containing barcodes for sample identification. Polymerase chain reactions (PCRs) were performed in triplicate using a total volume of 30 μL. Each reaction consisted of 4 μL of primers, 30 ng of DNA template, 25 μL of PCR Master Mix, and molecular biology-grade water, as needed. The following PCR thermocycling procedure was used: initial denaturation at 98 °C for 3 min; 30 cycles of 98 °C for 45 s, 55 °C for 45 s, and 72 °C for 45 s; and a final extension at 72 °C for 7 min. The PCR products were purified using Agencourt Ampure XP beads from Beckman Coulter, Inc. (Shanghai, China), and subsequently used for library construction. Finally, 250 bp paired-end sequencing of 80 samples was performed using the Illumina HiSeq/MiSeq platform at Shanghai Sangon Biotech (The equipment was purchased from Illumina, Inc. in San Francisco, CA, USA).

### 2.3. Sequence Filtering and Taxonomic Assignments

The raw image data generated from the Illumina MiSeq sequencer were processed using CASAVA software (version 1.8) for base recognition analysis, resulting in the conversion of images into original sequences, known as raw data or raw reads. These sequences were stored in FASTQ format. After removing primer and adapter sequences, the paired-end reads were merged into single sequences based on their overlap. Subsequently, the samples were identified and distinguished using barcode sequences, resulting in the acquisition of sample-specific data. Finally, the data collected for each sample were subjected to quality control and filtering processes to obtain valid data. USEARCH software (version 11) as used to eliminate unamplified sequence regions from the pretreated sequences, followed by error correction. Subsequently, UCHIME was utilized for the identification of chimeras [9,10]. Subsequently, we conducted BLASTn comparisons between the deleted chimeric sequences and representative sequences from the database. Sequences that fell below a specific alignment threshold were identified as sequences outside the target region and were subsequently removed because they were considered partial sequences.

### 2.4. Targeted Metabolomics of Feces

Concentrations of SCFAs in the rectum content were determined after water extraction (2 vol/wt) and protein precipitation using 10% (vol/vol) phosphotungstic acid (Sigma-Aldrich, Shanghai, China), as previously described [11]. The internal standard 2-ethylbutyrate (Sigma-Aldrich) was used for quantification. Samples were analyzed in duplicate. Data were collected and the peaks integrated using Open Lab Chem Station C.01.06 software (Agilent, Les Ulis, France). Data are expressed as μmol/g feces.

### 2.5. Statistical Analysis

To further investigate the impact of rearing mode on reproductive traits, a general linear model (GLM) in SAS (9.2) software was used to conduct ANOVA of the fixed effects [12]. The rearing modes were categorized into two types: AM and SM. The details of the specific model are as follows:

$$\gamma_{ij}=\mu +f_{i}+e_{ij}$$

In this equation, the phenotypic value of the reproductive traits is denoted as *Y_ij_*; *μ* represents the population mean; *f_i_* signifies a fixed effect; and *e_ij_* accounts for the random residual effect.

Community diversity within and between groups was assessed using multiple indices, including the observed species, Chao1 estimator, abundance-based coverage estimation (ACE), and Shannon and Simpson indices. All the indices were calculated using mothur software (1.48.1) [13]. The Wilcoxon rank-sum test was used to assess the differences in α diversity between the two groups, with a significance level of *p* < 0.05. Taxonomic comparisons between one or more samples at each classification level can be obtained using the results of the taxonomic analysis. Correlation analysis, a classical method for studying interactions between microorganisms, was also conducted. For this analysis, species or operational taxonomic units (ASVs) with an abundance exceeding 1% or ranking within the top 100 were selected for the bilateral test. The correlation coefficient and *p* value between each community/operational taxonomic unit (ASV) were calculated using SparCC [14]. The correlation matrix was plotted using the corrplot package in the R (4.3.0) programming language.

Phylogenetic measurements of β diversity were also performed using QIIME 2.0 [15]. The unweighted UniFrac distance was utilized for principal coordinate analysis (PCoA) to compare the microbial communities between the two groups. The unweighted UniFrac distance was utilized for principal coordinate analysis (PCoA) to compare the microbial communities between the two groups. Linear discriminant analysis effect size (LEfSe) was used for the identification and interpretation of biological markers and characteristics across multiple levels. This analysis utilized statistical methods to evaluate various characteristics of discovery and significance tests. The program initially employed the nonparametric Kruskal–Wallis (KW) rank-sum test to identify significant differences in abundances and characteristics between groups to ascertain associations among different groups or subgroups. Subsequently, a Wilcoxon rank-sum test (unpaired) was used to assess differences in the features between the groups. This served as a consistency check. Linear discriminant analysis (LDA) was subsequently performed to estimate the impact of group size differences. Functional enrichment analysis was conducted using PICRUSt to predict functional attributes based on the 16S rRNA gene sequencing data via the Greengenes database [16]. The significant differences between pairs of samples or multiple groups according to the Kyoto Encyclopedia of Genes and Genomes (KEGG) and Gene Ontology (GO) pathways were measured using STAMP (2.1.3) [17]. We used Pearson correlation analysis to examine the relationship between the fecal metabolome, the reproductive performance of Yorkshire pigs, and the gut microbiota.

## 3. Results

### 3.1. Analysis of Reproductive Performance in Pigs with Different Feeding Modes

Fixed-effect ANOVA plays a crucial role in statistics and experimental design, primarily being used to analyze differences between specific groups while controlling for other variables. Therefore, to assess the impact of different feeding regimes on the reproductive performance of Yorkshire sows, this study first conducted a fixed-effect ANOVA on the reproductive performance of the Yorkshire sows. The results show that the F values of NBD, NSB, TNB, and NBA are relatively large, indicating that the differences between groups are significant compared to within-group differences (*p* < 0.05). The F values for GL, NBW, MUM, LBW, and NW are relatively low, indicating that the between-group differences are smaller (*p* > 0.05) (Table 2).

On the other hand, the reproductive performance of sows is regulated by various factors, including nutrition, environment, and genetics. Therefore, to more accurately assess the differences between the two groups, we also analyzed the least squares means of the reproductive performance of Yorkshire sows under different feeding regimes. The results of the analysis showed that, in the AM group, both NBD and NSB were significantly greater than they were in the SM group (*p* < 0.05). Additionally, the TNB in the AM group was significantly lower than that in the SM group (*p* < 0.05). Notably, the NBA in the AM group was significantly lower than that in the SM group (*p* < 0.01) (Table 2).

### 3.2. Composition and Diversity of the Gut Microbiota

**Figure 1:**
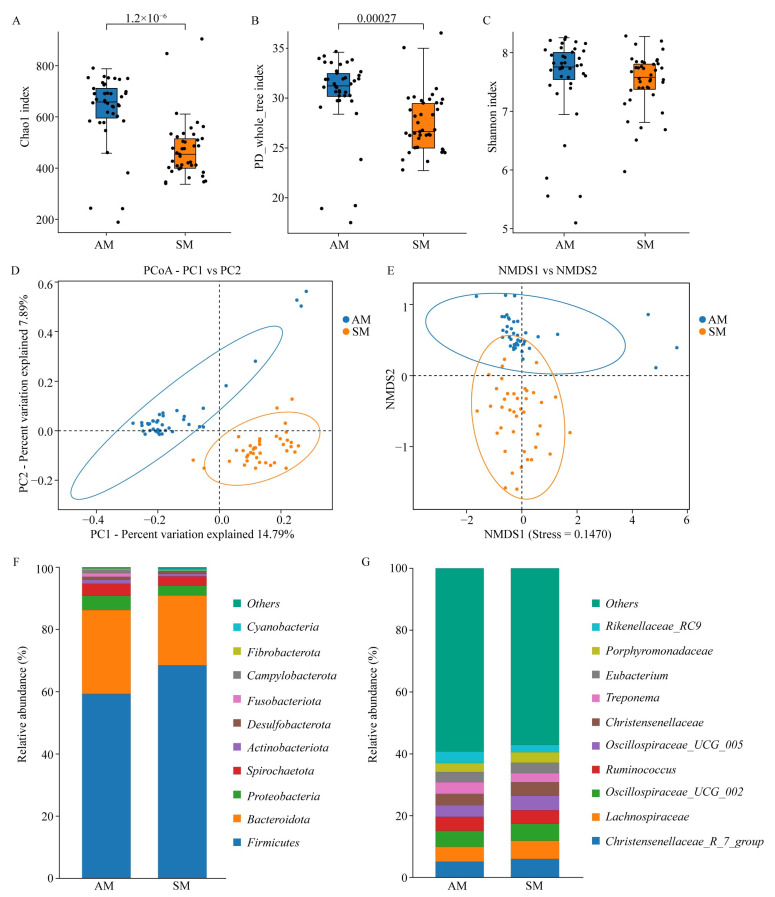
Diversity and composition of gut microbiota in Yorkshire pigs under different feeding modes. (**A**–**C**) Analysis of the α-diversity differences in the gut microbiota of Yorkshire pigs under different feeding modes; (**D**,**E**) Gut microbiota β-diversity analyzed using PCoA and NMDS clustering. (**F**,**G**) Distribution and abundance of the gut microbiota at the phylum and genus levels in Yorkshire pigs under different feeding modes. Note: AM: artificial feeding mode; SM: feeding station feeding mode.

In this study, 16S rRNA sequencing was conducted to analyze the intestinal microbiota of a total of 80 Yorkshire pigs raised under two feeding modes, AM and SM. A total of 21,173,318 read pairs were obtained, and after quality control and merging, 18,885,003 clean reads were generated. At least 64,410 clean reads were obtained for each sample, with an average of 236,063 clean reads. Subsequently, ASV clustering was performed based on a 97% similarity threshold. As a result, a total of 1552 ASVs were clustered in the AM group, while the SM group yielded 1503 ASVs. As commonly used methods in gut microbiota analysis, α-diversity and β-diversity analyses can jointly elucidate the complexity and differences within microbial communities and provide researchers with a comprehensive understanding of the overall composition of gut microbiota. Therefore, we conducted α-diversity and β-diversity analyses on the gut microbiota of Yorkshire sows under different feeding regimes. The results demonstrated significant elevations in both the Chao1 index and PD_whole_tree index in the AM group compared to those in the SM group (*p* < 0.05). This indicates that the richness of intestinal microbial species is higher in the AM group of sows, which harbors a more complex composition of gut microbiota. However, although the Shannon index of the AM group was significantly greater than that of the SM group, the difference was not significant (*p* > 0.05). This suggests that although the richness of species in the gut of the SM group is lower, the gut microbiota in the SM sows still maintains a stable community structure and ensures normal gut function (Figure 1A–C). Similarly, we conducted cluster analysis of the β diversity of the gut microbiota in Yorkshire pigs under different feeding modes using various methods, such as PCoA, NMDS, and heatmap analysis. The results are shown in Figure 1D,F, which demonstrate a clear separation between the gut microbiota of Yorkshire pigs under the artificial feeding mode and the feeding station feeding mode. The samples from the AM group are mainly clustered in the blue region at the top of the graph, while the samples from the SM group are primarily clustered in the orange region at the bottom. This indicates that there are significant differences in the gut microbiota structure between the AM and SM groups, with distinct mechanisms and interaction networks in their gut microbiota composition (Figure 1D,E).

The analysis of gut microbiota diversity revealed that the gut microbiota of Yorkshire pigs is primarily composed of phyla including *Firmicutes*, *Bacteroidota*, *Proteobacteria*, *Spirochaetota*, *Actinobacteriota*, *Desulfobacterota*, *Fusobacteriota*, *Campylobacterota*, *Fibrobacterota*, and Cyanobacteria, irrespective of the feeding mode. Specifically, *Firmicutes* and *Bacteroidota* emerged as the dominant phyla within the gut microbiota of Yorkshire pigs, accounting for more than 80% of the overall abundance of these bacteria. At the genus level, the gut microbiota of Yorkshire pigs under both feeding modes consisted of genera such as *Christensenellaceae_R_7_group, Lachnospiraceae*, *Oscillospiraceae_UCG_002*, *Ruminococcus*, *Oscillospiraceae_UCG_005*, *Christensenellaceae*, *Treponema*, *Eubacterium*, *Porphyromonadaceae*, and *Rikenellaceae_RC9* (Figure 1F,G).

### 3.3. Identification and Functional Prediction of Key Intestinal Flora That Affect Pig Reproductive Performance

**Figure 2:**
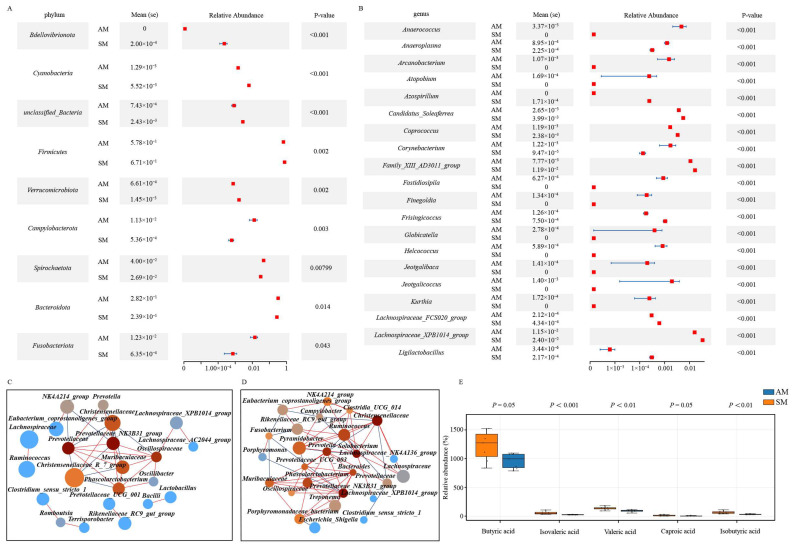
Microbiome and metabolome differential analysis. (**A**) There were significant differences in the abundance of microbial taxa at the phylum level in Yorkshire pigs under different feeding modes. (**B**) There were significant differences in the abundance of microbial taxa at the genus level in Yorkshire pigs among the different feeding modes. (**C**) Analysis of the intestinal microbiota interaction network in the AM group of Yorkshire pigs. (**D**) Analysis of the intestinal microbiota interaction network in the SM group of Yorkshire pigs. Note: The size of circles represents microbial abundance; the color depth represents microbial centrality; line colors indicate microbial correlations, where red indicates positive correlations and blue indicates negative correlations. (**E**) Analysis of differential short-chain fatty acids in feces of Yorkshire sows.

The analysis of differences in gut microbiome composition among different experimental groups has various applications and significance in biomedical research. It not only helps to explain the relationship between microbial composition and the host but also further elucidates the microbial pathways that contribute to phenotypic differences in the host. To assess the influence of various feeding modes on the abundance of gut microbiota in Yorkshire pigs, this study employed Metastats software 2.0 to conduct t tests on the species abundance data, revealing statistically significant disparities in microbial composition between the AM and SM groups. At the phylum level, there were significant differences in nine bacterial taxa between the AM and SM groups (*p* < 0.05). Similarly, the abundances of *Bdellovibrionota*, *Cyanobacteria*, *unclassified*_*Bacteria*, *Firmicutes*, and *Verrucomicrobiota* were significantly lower in the AM group than in the SM group (*p* < 0.05), while the abundances of *Campylobacterota*, *Spirochaetota*, *Bacteroidota*, and *Fusobacteriota* were significantly greater in the AM group than in the SM group (*p* < 0.05) (Figure 2A).

At the genus level, there were significant differences in the abundances of 106 bacterial taxa between the AM and SM groups (*p* < 0.05). Among them, the abundances of the bacterial taxa *Anaerococcus*, *Anaeroplasma*, *Arcanobacterium*, *Atopobium*, Corynebacterium, *Fastidiosipila*, *Finegoldia*, *Globicatella*, *Helcococcus*, *Jeotgalibaca*, *Jeotgalicoccus*, and *Kurthia* were significantly greater in the AM group than in the SM group (*p* < 0.05). Additionally, the abundance of *Azospirillum*, *Candidatus*_*Soleaferrea*, *Coprococcus*, *Family*_*XIII*_*AD3011*_*group*, *Frisingicoccus*, *Lachnospiraceae*_*FCS020*_*group*, *Lachnospiraceae*_*XPB1014*_*group*, and *Ligilactobacillus* taxa was significantly lower in the AM group compared to the SM group (*p* < 0.05) (Figure 2B) (Table S1). On the other hand, the construction of microbial interaction networks helps to reveal the interrelationships between microorganisms, facilitating the understanding of symbiotic or competitive relationships among microbial species. It also aids in uncovering the interactions between microorganisms in metabolic pathways of material exchange. Therefore, we also constructed the gut microbiome interaction networks of Yorkshire sows under different feeding regimes. The analysis of the microbial interaction network shows that the microbial interaction network in the SM group is more complex, with a greater number of core microbial species (genera that interact with five or more other species are defined as core microbes). *Prevotellaceae_NK3B31_group*, *Oscillospiraceae*, *Christensenellaceae*, *Muribaculaceae*, and *Phascolarctobacterium* are core microbial groups common to both feeding modes. *Prevotellaceae* and *Christensenellaceae_R_7_group* are core microbial groups unique to the AM group. *Solobacterium*, *Prevotella*, *Ruminococcus*, *Bacteroides*, *Pyramidobacter*, *Prevotellaceae_UCG_003*, *NK4A214_group*, *Porphyromonadaceae*_*bacterium*, *Clostridia_UCG_014*, and *Fusobacterium* are core microbial groups unique to the SM group (Figure 2C,D)

To identify the key microbial communities affecting the reproductive performance of Yorkshire sows, we further analyzed the correlation between the top 50 most abundant differential microbial species and the reproductive indicators of Yorkshire pigs. The analysis results indicated a significant positive correlation between *Christensenellaceae_R_7_group* and NBW, as well as between *unclassified_Atopobiaceae* and NBW, with correlation coefficients of 0.37 and 0.38, respectively (*p* < 0.05). Additionally, *unclassified_Muribaculaceae*, *unclassified_Atopobiaceae*, and *Pyramidobacter* each showed a significant negative correlation with GL, with correlation coefficients of −0.31, −0.28, and −0.36, respectively (*p* < 0.05). In contrast, *Fenollaria* exhibited a significant positive correlation with GL, with a correlation coefficient of 0.30 (*p* < 0.05). In the analysis of the correlation between other reproductive performance indicators such as NSB, MUM, TNB, and differential microbial communities, we also identified that *Clostridium_sensu_stricto_1*, *Romboutsia*, and *Turicibacter* were significantly positively correlated with NSB. Furthermore, *unclassified_Muribaculaceae*, *uncultured_rumen_bacterium*, and *Solobacterium* were each significantly positively correlated with LBW (*p* < 0.05). Finally, it is noteworthy that this study found a significant positive correlation between unclassified*_Atopobiaceae* and TNB, with a correlation coefficient of 0.27 (*p* < 0.05) (Figure S1).

**Figure 3:**
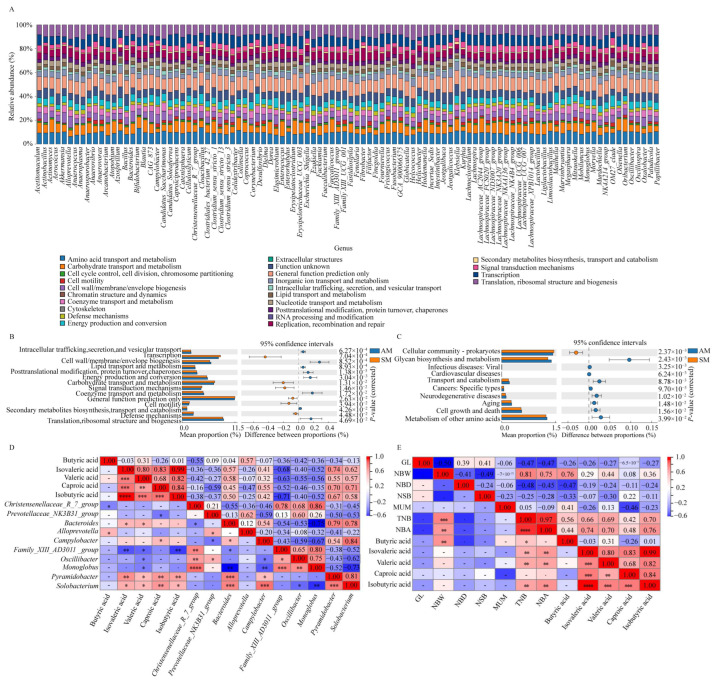
Functional prediction of differential intestinal flora in the two groups using GO and KEGG analyses. (**A**) KEGG enrichment analysis of the gut microbiota in late-pregnancy Yorkshire sows. (**B**) Functional analysis of differential gut microbiota constituents between Yorkshire sows in the AM and SM groups. (**C**) Differential pathway analysis of the gut microbiota in Yorkshire sows between the AM and SM groups. (**D**) Heat map of correlation coefficients between short-chain fatty acids and differential microbial communities. (**E**) Heat map of correlation coefficients between short-chain fatty acids and host reproductive performance. Note: An asterisk (*) indicates the correlation between two variables. (*, **, ***, ****) A higher number of asterisks represents a stronger correlation between the two variables, while fewer asterisks indicate a weaker correlation. No asterisk means there is no correlation between the two variables.

To delve into how feeding methods regulate host phenotypes by modulating gut microbiota structure, we performed enrichment and differential analysis of the functional pathways of gut microbiota in Yorkshire sows under different feeding regimes. This study aimed to perform an enrichment analysis of the functional categories and pathways associated with the gut microbiota in late-pregnancy Yorkshire sows using the GO and KEGG databases. The findings demonstrated that the functional categories and pathways enriched in the gut microbiota of Yorkshire late-pregnancy sows exhibited remarkable similarities, with predominant enrichment observed in the following categories: global and overview maps, carbohydrate metabolism, amino acid metabolism, metabolism of cofactors and vitamins, nucleotide metabolism, and translation (Figure 3A). The above results indicate that the gut microbiota of Yorkshire sows plays a similar role in the growth and reproductive processes of sows under different feeding regimes. Next, we analyzed the differences in the functionality and pathways of the gut microbiota induced by the different feeding modes. Figure 3B shows significant differences in the expression of the gut microbiota in late-pregnancy Yorkshire pigs in the AM and SM groups. Specifically, transcription, carbohydrate transport and metabolism, signal transduction mechanisms, general function prediction only, cell motility, and defense mechanisms exhibited significantly lower expression in the AM group than in the SM group (*p* < 0.05). Conversely, the AM group exhibited significantly higher expression in the categories intracellular trafficking, secretion, and vesicular transport, cell wall/membrane/envelope biogenesis, lipid transport and metabolism, post-translational modification, protein turnover, chaperones, energy production and conversion, coenzyme transport and metabolism, secondary metabolite biosynthesis, transport and catabolism, and translation, ribosomal structure and biogenesis when compared to the SM group (*p* < 0.05). The differential pathway enrichment analysis revealed that the expression of pathways related to the cellular community and prokaryotes was significantly lower in the AM group than in the SM group (*p* < 0.05). However, the expression of the pathways glycan biosynthesis and metabolism, infectious diseases: viral, cardiovascular diseases, transport and catabolism, cancers: specific types, neurodegenerative diseases, aging, cell growth and death, and metabolism of other amino acids was significantly greater in the AM group than in the SM group (*p* < 0.05) (Figure 3C).

### 3.4. Analysis of Fecal Differential Metabolites

Metabolic products, as crucial mediators of interactions between microbes and hosts, are involved not only in the host’s nutritional metabolism, signaling, and regulatory processes but also directly participate in the host’s energy metabolism, immune function, and neuroregulation. Short-chain fatty acids (SCFAs) are among the most important metabolites produced by the gut microbiota. Therefore, this study further measured the content of SCFAs in the feces of Yorkshire sows. The results showed that the levels of butyric acid and caproic acid in the feces of sows in the SM group were significantly higher than those in the AM group (*p* < 0.05), while the levels of isovaleric acid, valeric acid, and isobutyric acid were extremely significantly higher than those in the AM group (*p* < 0.01). There was no significant difference in the levels of acetic acid and propionic acid between the two groups (Figure 2E).

To verify the impact of the gut microbiota-metabolic product pathway on the host’s reproductive performance, this study further analyzed the correlation between key gut microbiota, differential short-chain fatty acids, and host reproductive performance indicators. The heatmap analysis results showed that butyric acid had a strong correlation with NBW and TNB, with correlation coefficients of 0.76 and 0.56, respectively. Isovaleric acid, valeric acid, and isobutyric acid all had a strong positive correlation with TNB and NBA, with correlation coefficients all above 0.66 (Figure 3E). On the other hand, the heatmap of the interaction between key gut microbiota and short-chain fatty acids showed that *Bacteroides*, *Pyramidobacter*, *Solobacterium*, *Campylobacter*, and *Alloprevotella* all had a strong positive correlation with various short-chain fatty acids, with correlation coefficients all greater than 0.32. It is worth noting that among them, *Solobacterium* and *Pyramidobacter* are core microbiota unique to the SM group. *Christensenellaceae*_*R_7_group*, *Prevotellaceae*_*NK3B31_group*, *Family_XIII_AD3011_group*, *Oscillospiraceae*, and *Monoglobus* all had a strong negative correlation with various short-chain fatty acids, with *Christensenellaceae_R_7_group* being the core microbiota unique to the AM group (Figure 3D,E). Therefore, based on the above analysis results, we further elucidated the pathways through which key microbial communities affect Yorkshire sows.

## 4. Discussion

It is well known that the gut microbiota plays a crucial role in nutrient absorption in the host. Studies have shown that 35% of the enzymes required for intestinal digestion are derived from the microbiota, with 25% of these active enzymes involved in carbohydrate metabolism [18]. Additionally, research has found that the high abundance of Escherichia (Escherichia Castellani and Chalmers) and Brucella in the ileum contributes to the degradation of glucose and fructooligosaccharides [19,20], while Actinomycetes in the cecum promote polysaccharide fermentation [21]. In the colon, Lactobacillus (Bacterium lactis) and Streptococcus contribute significantly to lactic acid production [22]. Beyond carbohydrates, numerous studies have highlighted the crucial role of the gut microbiota in host protein metabolism and nutrient utilization [23,24]. For instance, Succinivibrionaceae, Klebsiella, Escherichia coli, and Streptococcus, which are primarily involved in protein metabolism in the small intestine, have the ability to secrete various proteases and peptidases that directly metabolize amino acids [25]. In monogastric animals, Prevotella Shan and Collins, Clostridium butyricum, and Streptococcus bovis secrete highly active dipeptidyl peptidases and peptidases for protein digestion and absorption.

In addition to playing an important role in nutrient absorption in the host, the gut microbiota also plays an equally important role in maintaining the host’s health. Gut homeostasis is a dynamic equilibrium formed by the interaction between the intestinal barrier and the internal environment. Any imbalance in any element of gut homeostasis may lead to host disease [26]. Research has found that microorganisms adhering to the intestinal mucosa can form a bacterial membrane barrier, in which the S-layer protein of lactobacilli can specifically adhere to the host intestinal epithelial cells, thereby protecting the intestinal barrier, preventing the invasion and colonization of pathogenic bacteria16. Moreover, the surface layer protein A of Lactobacillus acidophilus can adhere to colorectal cancer-related cells, induce the expression of tight junction protein ZO-1, enhance tight junctions, and reduce cell permeability [27,28]. In addition, gut microbiota can also reduce the colonization of pathogenic bacteria by producing metabolic products such as fatty acids and organic acids to lower the gut pH, promote intestinal peristalsis, and strengthen the competitive advantage of probiotics. For example, the phosphoenolpyruvate produced by bifidobacteria can specifically bind to intestinal epithelial cells to produce glycosidases that degrade oligosaccharides, thereby inhibiting the colonization of pathogens. Furthermore, gut microbiota can also act as an immune barrier by interacting with the host’s immune system. For example, the flagella, pili, and capsules of microorganisms themselves can serve as antigens to induce the body to produce an immune response [29]. Given the critical role of the gut microbiota in host growth and survival, we investigated the pathways through which environmental-induced variations in gut microbiota affect host reproductive performance. In this study, we analyzed the relationship between the composition of the intestinal flora and the reproductive performance of Yorkshire sows during late pregnancy under different feeding patterns and explored the effects of different feeding patterns on reproductive performance from the perspective of the intestinal flora. The results of this study indicate that different feeding methods have a significant impact on reproductive performance indicators such as the TNB and NBA in the same breed of pigs. SM effectively enhanced reproductive performance indicators such as TNB and NBA in Yorkshire pigs compared to AM. In addition, the analysis of the gut microbiome composition indicates that regardless of the feeding method used, the gut microbiota of Yorkshire pigs is primarily composed of *Firmicutes* and *Bacteroidetes*. However, there are some differences in the abundance of different microbial communities, which is consistent with the findings of previous studies [30,31].

Gut microbiota maintains relative stability, evidenced by the similarity in microbial composition across different periods in an individual being consistently higher than in other individuals, while also being dynamically influenced by environmental changes [32]. The relative stability and dynamic adjustability of the gut microbiota play a crucial role in host adaptation and health. Studies have shown that factors such as diet, host living environment, antibiotic use, and host genetics significantly impact gut microbiota composition. Diet is one of the primary drivers of gut microbiota composition and variation. In a study by Zhang et al. on mice with different dietary regimens, it was found that diet explained 57% of the variation in gut microbiota composition [33]. The composition, quantity, and ratio of the three macronutrients—carbohydrates, proteins, and fats—significantly influence gut microbiota composition. Different gut microbes metabolize various nutrients with differing energy efficiencies. Bacteria in the colon can produce short-chain fatty acids by degrading indigestible polysaccharides such as fibers and pectins found in plant cell walls, which in turn alters the pH of the gut environment, affecting gut microbiota composition. Carbohydrates in food play a crucial role in maintaining the stability of gut microecology. Non-digestible oligosaccharides in the diet can significantly promote the proliferation of bifidobacteria in piglets, thereby competitively inhibiting the growth of Escherichia coli. Research by Haenen et al. also indicated that resistant starch in food can regulate gut microbiota composition and the concentration of short-chain fatty acids [34]. Antibiotics are commonly used in animal production for disease prevention and control, but they also have a substantial impact on gut microbiota composition, particularly in piglets, where their regulatory effects primarily occur in the stomach and small intestine. Studies have shown that the intervention of a combination of oxytetracycline and quinocetone can significantly reduce the abundance of Lactobacillus species in piglets while increasing the relative abundance of the potential pathogen Streptococcus suis. Additionally, gut microbiota composition is influenced by living environments. For instance, Davenport et al. found that the gut microbiota of humans living in a primitive lifestyle fluctuates with seasonal changes, while Worthmann et al. discovered that cold temperatures can convert cholesterol to bile acids, thereby altering the gut microbiota in mice [35,36].

It is well known that the gut microbiota plays a crucial regulatory role in the metabolism and absorption of nutrients in the host. In recent years, with the continuous improvement of sequencing technologies, there has been a deeper understanding of gut microbiota characteristics. Regarding fat metabolism, studies have found that the microbiota has a significant impact on host health, such as obesity and fat deposition. Hildebrandt et al. suggested that the high fat content in the diet is the cause of gut microbiota imbalance in obese patients [37,38]. Other researchers have also found that diseases such as gut inflammation, insulin resistance, type 2 diabetes, and fatty liver are caused by an increase in Gram-negative bacteria in the gut due to a high-fat diet, leading to elevated levels of metabolic endotoxins such as lipopolysaccharides and impaired gastrointestinal barrier function [39,40]. In animal populations, Velagapudi et al. compared the lipid metabolism profiles of conventional mice and germ-free mice and found that the levels of energy metabolites such as acetate, citrate, fumarate, and succinate were elevated in conventional mice, while cholesterol and fatty acid levels were reduced, and the lipid clearance rate was increased. This indicates that the gut microbiota can participate in host energy and lipid metabolism [41]. Estradiol is an important hormone that affects the reproductive performance of the host. Research on estradiol indicates that it plays a role in promoting proliferation and repair of the endometrium, exerting a wide-ranging impact on the uterus and being closely related to the reproductive performance of female animals. However, elevated levels of estradiol may reduce the reproductive performance of female animals and increase their susceptibility to diseases [42,43,44]. Shin et al.‘s study found that the abundance of *Bacteroidota* and *Firmicutes* is correlated with estradiol levels in the host. The group with a higher abundance of *Bacteroidota* had higher levels of estradiol in the host’s body. In this study, we found that the abundance of *Bacteroidota* in the intestines of the AM group was significantly higher than that in the SM group (*p* < 0.05). This suggests that the AM group of Yorkshire pigs may have higher estradiol levels in their bodies, which could be a potential reason for the reduced reproductive performance observed in the AM group [45].

The reproductive performance of sows is related to the composition and structure of fecal microbiota. The intestinal microbiota diversity of high-reproductive-performance Meishan sows is significantly higher than that of low-reproductive-performance Meishan sows. Furthermore, there are significant differences in the dominant microbial communities in the feces of high-reproductive-performance sows (litter size > 15) and low-reproductive-performance sows (litter size ≤ 7) during late pregnancy and lactation [46,47]. Multiple studies have shown that the abundance of Streptococcus, a genus of bacteria, is lower in the feces of high-reproductive-performance sows. Streptococcus is a major pathogen that leads to decreased production performance and increased mortality in pigs, thereby affecting litter size in sows [46,48]. Research has found that transplanting fecal microbiota from Meishan gilts into the intestines of Meishan sows can promote follicular development and steroid hormone metabolism in sows. Transplantation enriches the fecal microbiota of sows with *Ruminococcus*_*bromii*, *Bifidobaterium*_*thermophilum*, *Ruminococcus*_flavefaciens, *Fibrobacteres*, *Lactobacillus*_reuteri, and *Fibrobacteres,* thereby increasing the diversity and abundance of the intestinal microbiota in gilts. Similarly, there is a close relationship between estrus and the gut microbiota in sows. After weaning, there are differences in the dominant microbial communities in the feces of sows in estrus and non-estrus. Probiotics can shorten the weaning-to-estrus interval. In studies by Inatomi et al., feeding with a probiotic compound consisting of *Lactobacillus* acidophilus, *Butyrivibrio fibrisolvens*, and Clostridium butyricum from 6 weeks before parturition to 7 days postpartum significantly reduced the weaning-to-estrus interval. Similarly, adding Bacillus subtilis C-3102 to the feed also shortened the weaning-to-estrus interval in sows [49,50]. Therefore, based on the above research findings, we conducted an assessment of the gut microbiota structure in Yorkshire sows. At the genus level, research has demonstrated that the effects of diet-induced gut microbiota dysbiosis extend beyond the gut. Zhang et al. showed that a decrease in the abundance of *Ruminococcaceae_NK4A214_group* in the gut can lead to abnormal metabolism of vitamin A in the gut, thereby causing abnormal sperm production and impacting the reproductive performance of the host [51]. Moreover, considering maternal microbial transfer, the gut microbiota is significantly linked to the reproductive performance of the host. Studies indicate that during fetal development, compounds produced by the maternal microbiota are transferred to the fetus and offspring [52]. This transfer enhances the generation of fetal innate immune cells, thereby contributing to the reproductive performance of the host, with *Escherichia coli* playing a significant role in this process. Hence, the genus-level microbial community plays a pivotal role in the host reproductive process [52,53]. Consequently, we analyzed the variations in the genus-level gut microbiota of Yorkshire pigs during late pregnancy under different feeding regimens. At the genus level, there were significant differences in the abundances of 106 bacterial taxa between the AM and SM groups (*p* < 0.05). The AM group exhibited significantly greater abundances of *Corynebacterium* and *Parabacteroides* than the SM group (*p* < 0.05) did. Previous research has indicated a correlation between *Corynebacterium* and *Parabacteroides* and decreased levels of follicular estradiol and luteinizing hormone, suggesting that these hormones may impact the reproductive performance of Yorkshire pigs by influencing their levels [54]. The findings of pig research suggest that reducing the abundance of *Fusobacterium* can enhance sows’ immune performance and, to some degree, improve their reproductive performance. Moreover, their research findings indicate that the proliferation of beneficial bacteria, including *Lachnospiraceae*_*XPB1014_group* and *Roseburia*, can notably enhance the daily feed intake of pregnant sows, promote the growth rate of weaned piglets, lower the fecal moisture content of sows, decrease the incidence of constipation in sows, and elevate the levels of anti-inflammatory factors in the sows’ intestines [19,20]. In this study, Yorkshire sows in the SM group demonstrated superior reproductive performance. Additionally, the analysis of intestinal flora diversity revealed a significantly greater abundance of beneficial bacteria, including *Lachnospiraceae_FCS020_group*, *Lachnospiraceae_XPB1014_group*, *Lachnospiraceae_AC2044_group*, *Lachnospiraceae_NK4A136_group*, *Lachnospiraceae_UCG_007* and *Roseburia*, in the sows’ intestines than in those of the AM group. These results align with those of prior research [6,55]. Overall, the aforementioned research results demonstrated that the feeding method impacts the composition of the sow’s intestinal microbiota. These variations in the gut microbiota can regulate the host’s reproductive performance by influencing hormone levels and nutrient absorption [56]. Additionally, probiotics can enhance the immune indices of weaned piglets through probiotic transfer, thereby improving piglet survival rates [57].

Next, we constructed an intestinal microbial interaction network for Yorkshire sows and identified core microbiota that regulate the abundance of multiple bacterial groups. Of particular note, the *Christensenellaceae_R_7_group* is a unique core species in the intestines of AM group Yorkshire sows, and it plays a regulatory role in the abundance of various bacterial groups. Research has shown a positive correlation between *Christensenellaceae_R_7_group* and maternal placental oxidation and inflammatory responses, which can have a certain impact on the reproductive performance of the host, although the exact mechanism of its action is not yet clear [58]. Similarly, *Pyramidobacter*, *Solobacterium*, and *Bacteroides* are core intestinal microbiota unique to the SM group, and each is significantly associated with the abundance of various intestinal bacterial groups. Research has shown that *Pyramidobacter* is significantly negatively correlated with fasting blood glucose in late-term pregnant women, effectively inhibiting the occurrence of gestational diabetes and improving the reproductive performance and health status of the host [59]. Additionally, studies have indicated that *Solobacterium* is associated with host hormone secretion and reproductive performance, showing a positive correlation between its abundance and pregnancy success rates [60]. These studies collectively demonstrate the beneficial effects of core microbiota in the SM group on the reproductive performance of the host. However, the specific mechanisms and pathways through which they operate remain unclear. Therefore, our subsequent research includes pathway and functional enrichment analysis of intestinal microbiota, as well as targeted metabolomics of intestinal contents, to preliminarily investigate the pathways and mechanisms through which intestinal microbiota influence host phenotypes.

Numerous studies have found that the intestinal microbiota can affect the reproductive performance of sows through various pathways, such as hormone levels and host metabolism. Research has shown that the reproductive cycle of sows is strictly regulated by hormones. Luteinizing hormone can induce pre-ovulatory follicle development and ovulation, promoting the formation of the corpus luteum and the secretion of progesterone [61]. Follicle-stimulating hormone and luteinizing hormone jointly regulate the secretion of estrogen in the host. From early pregnancy to late pregnancy, plasma prolactin, progesterone, follicle-stimulating hormone, and estrogen levels decrease [62]. Estrogen and progesterone play a crucial role in the development of the endometrial glands, with estrogen inducing proliferation of the uterine cavity epithelium and glandular epithelium [63]. Numerous studies have found that the intestinal microbiota interacts with reproductive hormones in sows, and the gut microbiota can directly or indirectly regulate the levels of reproductive hormones, thereby affecting the reproductive performance of sows. Some bacteria, such as Bacteroides, unclassified *Lachnospiraceae*, *Clostridiales*, and *Akkermansia*, can metabolize estrogen and progesterone, which in turn promote the growth of Bacteroides [62]. Additionally, leptin and insulin are also regulated by the intestinal microbiota. Leptin is mainly involved in reproductive regulation through receptors in the hypothalamus, pituitary gland, ovaries, and endometrium [64,65]. To elucidate the impacts of different feeding methods on the reproductive performance of Yorkshire pigs from a microbiota perspective, we conducted GO and KEGG enrichment analyses to identify the pathways and functions associated with different microbiota resulting from various feeding methods [66,67]. The findings demonstrated that the functional categories and pathways enriched in the gut microbiota of late-pregnancy Yorkshire sows exhibited remarkable similarities, with predominant enrichment observed in the global and overview maps, carbohydrate metabolism, amino acid metabolism, metabolism of cofactors and vitamins, nucleotide metabolism, and translation. The functional enrichment terms were enriched in general function prediction only; amino acid transport and metabolism; translation, ribosomal structure and biogenesis; transcription, carbohydrate transport and metabolism; cell wall/membrane/envelope biogenesis; replication, recombination and repair; and energy production and conversion. Specifically, the transcription, carbohydrate transport and metabolism, signal transduction mechanism, general function prediction only, cell motility, and defense mechanism categories exhibited significantly lower expression in the AM group than in the SM group (*p* < 0.05). These findings indicate that while artificial feeding allows for precise control of the feeding amount and duration based on animal needs and conditions, it also introduces additional pathogenic bacteria. Artificial feeding increases the diversity and richness of the intestinal flora in late pregnant sows but diminishes energy metabolism and immune capacity, consequently affecting reproductive performance. Pathway enrichment analysis of the AM group revealed a significantly greater enrichment of infectious diseases, cardiovascular diseases, and neurodegenerative diseases than that of the SM group, further confirming our hypothesis.

Intestinal microorganisms ferment dietary fiber and resistant starch into short-chain fatty acids, mainly including acetic acid, propionic acid, and butyric acid, which participate in energy homeostasis regulation, maintain intestinal homeostasis, regulate immunity, and have anti-inflammatory effects [68,69]. Bacteroides mainly produce acetic acid and propionic acid, *Firmicutes*, *Ruminococcaceae*, and *Lachnospiraceae* mainly produce butyric acid, and *Christensenellaceae* can produce acetic acid and butyric acid. Common Bacteroides and *Prevotella* can produce propionic acid through the succinic acid pathway [70]. Butyric acid is metabolized by host colon cells, providing 60% to 70% of the energy for colon cells, which helps to improve fetal growth and increase reproductive performance in sows [71]. In pregnant mice, SCFAs in the colon can provide energy for the embryos and maintain postnatal energy metabolism through the SCFA-GPR41 and SCFA-GPR43 axes [72]. SCFAs can inhibit the transcription of the PRL gene, reduce PRL production, and also promote the release of gastrointestinal hormones and increase sex hormone levels through the brain–gut axis [73]. To further validate our hypothesis, we conducted targeted metabolomic analysis of the intestinal contents of Yorkshire sows in order to elucidate the impact of the gut microbiota on host reproductive performance through microbial–metabolite pathways. The results showed significant differences in the concentrations of butyric acid, caproic acid, isovaleric acid, valeric acid, and isobutyric acid between the AM and SM groups *(p* < 0.05). Previous studies have indicated that butyric acid has garnered extensive attention and research in animal husbandry, as it can enhance the fertilization rate and reproductive performance of sows by promoting follicular development and progesterone secretion. Additionally, it can improve reproductive performance by influencing cortisol secretion during pregnancy and childbirth. Furthermore, although isovaleric acid, valeric acid, and isobutyric acid do not directly regulate host reproductive performance, they are important energy sources and components of cell membranes within the animal body, potentially affecting physiological functions and reproductive capabilities. Adequate short-chain fatty acids contribute to maintaining the fatty acid balance within the animal, thus supporting normal reproductive functions [74,75,76]. The above conclusions further support the results of this study to a certain extent. Through the analysis of metabolomic data and its correlation with host reproductive performance, this study also identified short-chain fatty acids significantly correlated with host total number born (TNB) and number born alive (NBA), indicating that short-chain fatty acids are indeed a pathway regulating host phenotypes. To validate the relationship between short-chain fatty acids and gut microbiota, we conducted a correlation analysis between microbiota and short-chain fatty acids. The results revealed that *Christensenellaceae_R_7_group*, a core microbiota unique to the AM group, exhibited significant positive correlations with *Family_XIII_AD3011_group*, *Oscillibacter*, and *Monoglobus*, while all four microbial abundances showed significant negative correlations with short-chain fatty acids. Conversely, *Pyramidobacter*, *Solobacterium*, and *Bacteroides*, as core microbiota specific to the SM group, exhibited significant positive correlations with short-chain fatty acids. These findings further demonstrate that different feeding methods lead to variations in the gut microbiota, which, through metabolic pathways, influence host phenotypes, thereby resulting in differences in host reproductive performance.

## 5. Conclusions

Obviously, with the advancement of technology, automated feeding systems are gradually replacing manual feeding methods. Through precise sensors and systems, automated feeding stations can more effectively monitor the status of sows, improve animal health and welfare by measuring environmental, physiological, and behavioral variables, and consequently enhance the production and reproductive performance of sows. This study systematically investigated sows under different feeding modes, starting from differences in reproductive performance, to explore the impact of feeding methods on host gut microbiota and host metabolism. This study identified 106 differential microbial taxa resulting from different feeding modes and further screened 10 key taxa significantly associated with the reproductive performance of Yorkshire sows: *Christensenellaceae_R_7_group*, *unclassified_Muribaculaceae*, *unclassified_Atopobiaceae*, *Clostridium_sensu_stricto_1*, *uncultured_rumen_bacterium*, *Romboutsia*, *Bacteroides*, *Turicibacter*, and *Pyramidobacter*. Subsequently, the correlation between the metabolome, host phenotype, and gut microbiota further confirmed the pathway through which gut microbiota influences the host phenotype via short-chain fatty acids such as acetate, propionate, and butyrate. These research findings provide a theoretical basis and data support for the subsequent promotion of automatic feeding systems and feeding modes, and may contribute to the formulation of strategies to improve the reproductive performance of sows.

## Figures and Tables

**Table 1:** Composition and nutritional levels of the base diet.

Diet Composition	Content /%	Level of Nutrition	Content/%
Corn	63.10	Metabolizable Energy/MJ·kg^−1^	14.23
Soybean meal (43%)	25	Crude protein	17.8
Bran	4	Total lysine	1.03
Choice white grease	2	Ca	0.71
Fish meal (67%)	2	Total phosphorus	0.56
Sow Vit-Min premix	0.50	Effective phosphorus	0.34
Salt	0.40		
Dicalcium phosphate	0.90		
Limestone	0.80		
Lysine	0.15		
Methionine	0.05		
Threonine	0.03		
Tryptophan	0.02		
Choline chloride	0.1		
Zeolite powder	0.95		
Total amount	100		

Note: The premix provides, per kilogram of feed, vitamin A, 10,000 IU; vitamin D3, 1800 IU; vitamin E, 100 IU; vitamin K3, 4.5 mg; vitamin B1, 2.0 mg; riboflavin, 6.0 mg; vitamin B6, 7.0 mg; vitamin B12, 0.05 mg; niacin, 30 mg; pantothenic acid, 35 mg; folic acid, 3.5 mg; biotin, 0.5 mg; choline chloride, 500 mg; iron, 80 mg; copper, 20 mg; zinc, 100 mg; manganese, 25 mg; iodine, 0.14 mg; and selenium, 0.15 mg.

**Table 2:** Analysis of variance and least squares means for the fixed effects of reproductive traits in Yorkshire sows under different feeding regimes.

Trait	Feeding Modes				*p*-Value
	df	F-Value	AM	SM	
GL	1	0.05	117.33 ± 1.73	117.15 ± 1.51	0.8301
NBW	1	0.05	1.39 ± 1.52	1.45 ± 1.60	0.8270
NBD	1	5.22 *	0.06 ± 0.35	0.04 ± 0.28	0.0223
NSB	1	5.43 *	1.83 ± 2.09	1.42 ± 1.48	0.0199
MUM	1	0.56	0.45 ± 1.00	0.39 ± 0.87	0.4549
TNB	1	4.40 *	17.38 ± 4.27	17.53 ± 4.30	0.0364
NBA	1	11.84 **	13.71 ± 3.50	14.24 ± 3.41	0.0006
LBW	1	0.86	18.15 ± 4.92	18.85 ± 4.67	0.3585
NW	1	0.06	11.48 ± 4.72	12.38 ± 4.64	0.8143

Note: * represents significance (*p* < 0.05); ** represents extreme significance (*p* < 0.01). Explanation of abbreviations: TNB: total number born, NBA: number born alive, LBW: litter birth weight, GL: gestation length, NW: number of weaned piglets, NBW: number of piglets born weak, NSB: number of stillbirths, MUM: number of mummies, NBD: number of birth deformities. All phenotypic data in this table are presented as mean ± standard deviation.

## Data Availability

The datasets presented in this study can be found in online repositories. The names of the repository and accession number(s) can be found below: Sequence Read Archive (NCBI, USA), PRJNA1157551.

## Supplementary Materials

The following supporting information can be downloaded at: https://www.mdpi.com/article/10.3390/ani14182714/s1.
