# Supplementary figures and images for: Effects of Interactions between Feeding Patterns and the Gut Microbiota on Pig Reproductive Performance
Source: Animals (Basel). 2024 Sep 19;14(18):2714. doi: 10.3390/ani14182714 (PMC11428678; doi:10.3390/ani14182714)

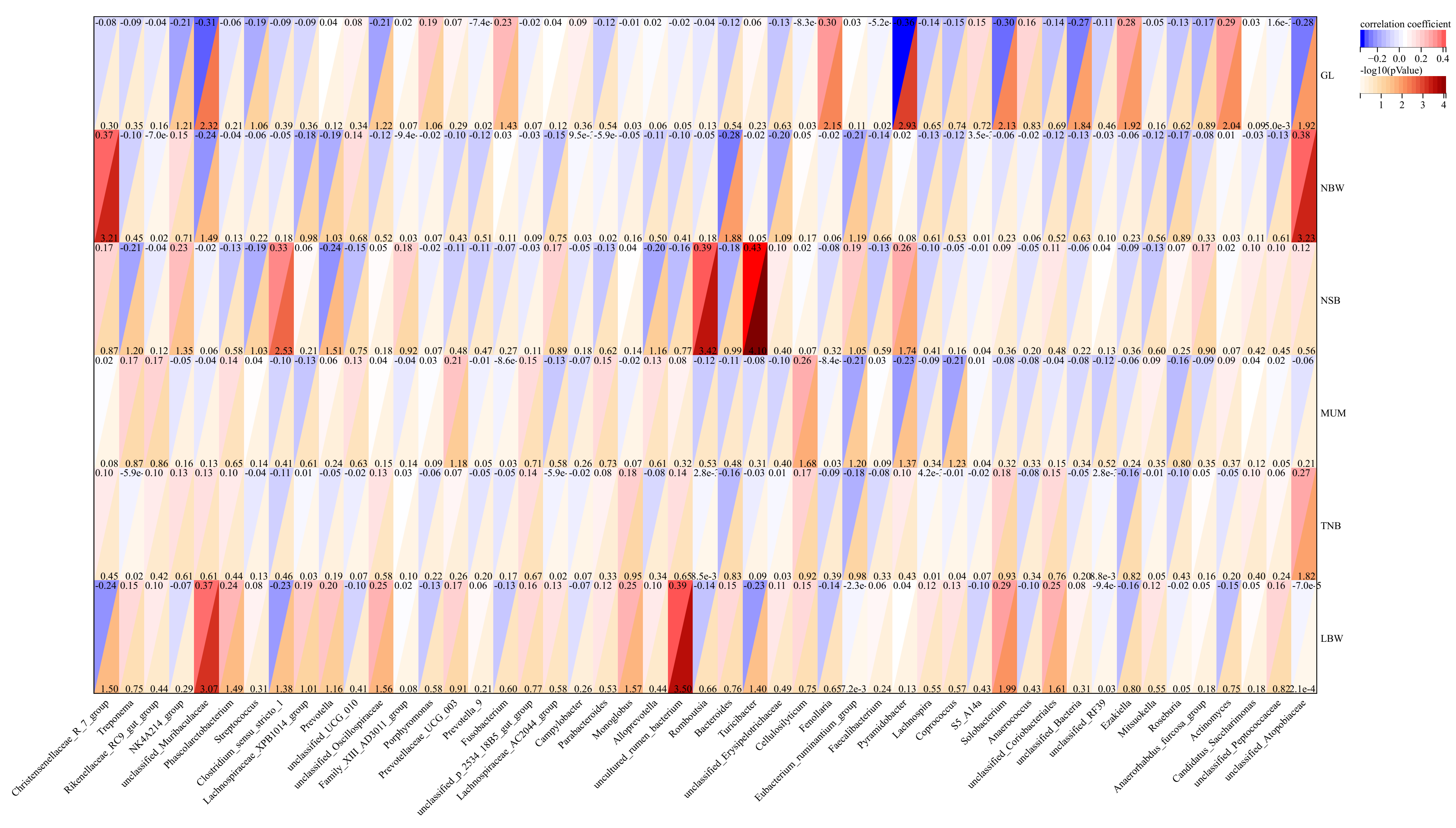

Supplement: Supplementary file 1 [file animals-14-02714-s001.zip › Figure S1.pdf]
